# The emerging role of m^6^A modification of non-coding RNA in gastrointestinal cancers: a comprehensive review

**DOI:** 10.3389/fcell.2023.1264552

**Published:** 2023-10-30

**Authors:** Meiqi Wang, Zhuo Liu, Xuedong Fang, Xianling Cong, Yue Hu

**Affiliations:** ^1^ Department of Gastrointestinal Colorectal and Anal Surgery, China-Japan Union Hospital of Jilin University, Changchun, China; ^2^ Department of Biobank, the China-Japan Union Hospital of Jilin University, Changchun, China; ^3^ Department of Dermatology, China-Japan Union Hospital of Jilin University, Changchun, China

**Keywords:** m^6^A, mRNA, lncRNA, esophageal cancer, gastric cancer, colorectal cancer

## Abstract

Gastrointestinal (GI) cancer is a series of malignant tumors with a high incidence globally. Although approaches for tumor diagnosis and therapy have advanced substantially, the mechanisms underlying the occurrence and progression of GI cancer are still unclear. Increasing evidence supports an important role for N^6^-methyladenosine (m^6^A) modification in many biological processes, including cancer-related processes via splicing, export, degradation, and translation of mRNAs. Under distinct cancer contexts, m^6^A regulators have different expression patterns and can regulate or be regulated by mRNAs and non-coding RNAs, especially long non-coding RNAs. The roles of m^6^A in cancer development have attracted increasing attention in epigenetics research. In this review, we synthesize progress in our understanding of m^6^A and its roles in GI cancer, especially esophageal, gastric, and colorectal cancers. Furthermore, we clarify the mechanism by which m^6^A contributes to GI cancer, providing a basis for the development of diagnostic, prognostic, and therapeutic targets.

## 1 Introduction

**FIGURE 1:**
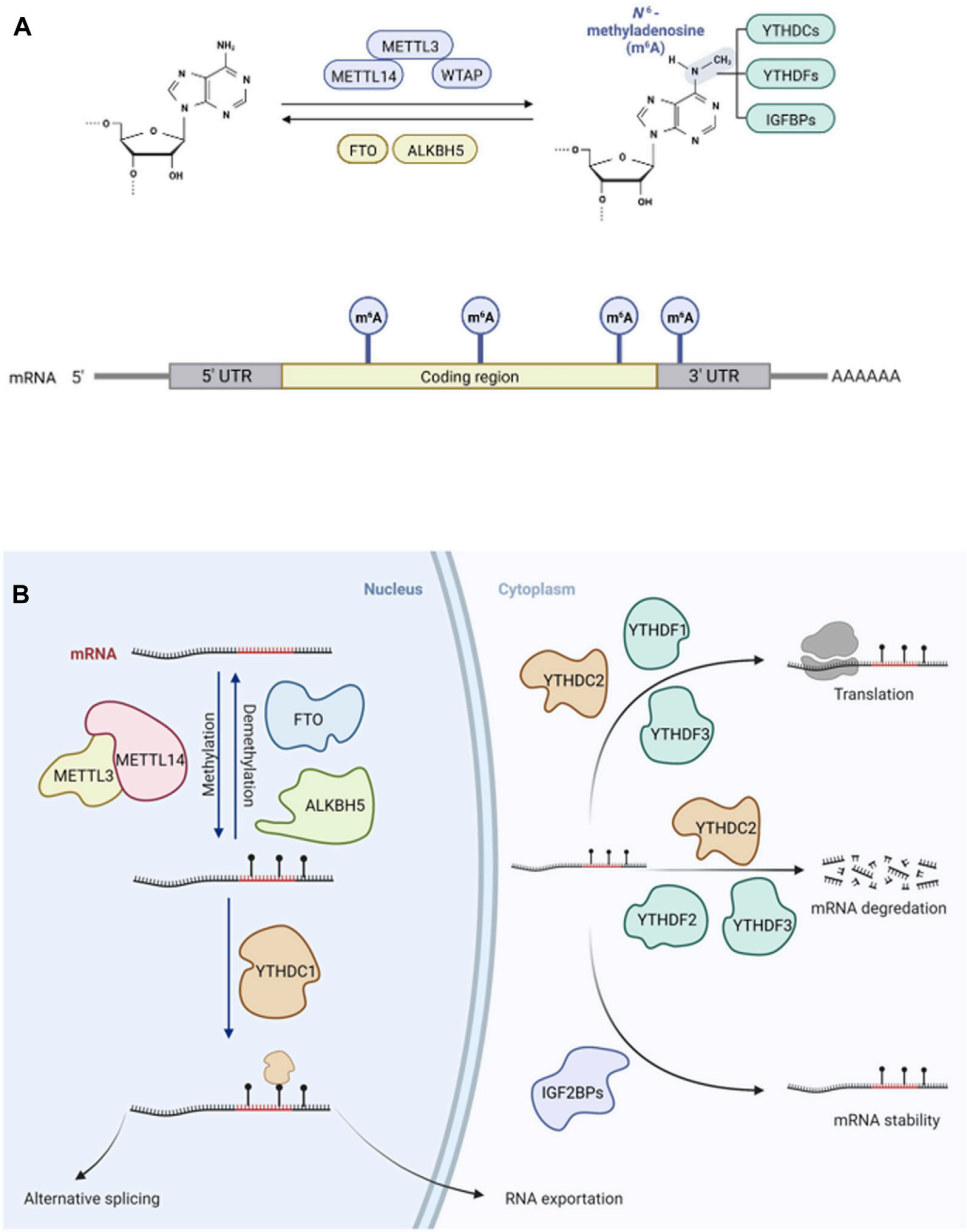
Dynamic and reversible m^6^A modification on mRNA in eukaryocytes. **(A)**. The adenosine **(A)** can be methylated by m^6^A “writer”, a complex of METTL3, METTL14 and WTAP, becoming N6-methyladenosine (m^6^A) and m^6^A can be demethylated by m^6^A “erasers”, FTO and ALKBH5 reversibly. m^6^A can be recognized by m^6^A” readers”, influencing targeted mRNA fates. m^6^A is mostly deposited on exons of 3′ untranslated regions (UTRs) and stop codons of mRNA. **(B)**. The modification of m^6^A “writers”, “erasers” and “readers” can lead to aberrant regulations in cancers, such as RNA exportation, splicing, translation, degradation, and stability.

Gastrointestinal (GI) cancer is a set of malignant tumors accounting for over 25% of cancer incidence annually, and the morbidity and mortality of esophageal, gastric, colon, rectal, liver, pancreatic, and gallbladder cancers rank high with an estimated 5 million new cases and 3,609,607 deaths in the global cancer statistics for 2020 (Sung et al., 2021). GI cancer poses a serious threat to human health with a large number of cases. Due to the growth of population, aging, and lifestyle changes, the burden of digestive system tumors worldwide especially in East Asia is increasing day by day, and the incidence of tumors is becoming younger. Despite advances in immunotherapy and molecular targeted therapy in addition to conventional surgery, radiotherapy, and chemotherapy, the early diagnosis and treatment of advanced GI tumors are still unsatisfactory owing to an incomplete understanding of the molecular mechanisms. Accordingly, it is necessary to further clarify the mechanisms underlying these digestive tract cancers.

m^6^A (N^6^-methyladenosine) modification has a significant regulatory role in many biological processes and diseases (Frye et al., 2018; Wei and He, 2021). Recent studies have demonstrated that m^6^A plays an important role in tumor progression and suppression, especially in acute myeloid leukemia (AML), glioblastoma (GBM), and breast cancer (Table 1). Vu et al. found that m^6^A could control the translation of *PTEN*, *c-MYC*, and *BCL2*, which are involved in the differentiation of hematopoietic stem/progenitor cells in AML (Vu et al., 2017). m^6^A modifications have also been linked to survival rate in GBM, in which interactions with long non-coding RNAs (lncRNAs) have been demonstrated (Zhang et al., 2017). In breast cancer, m^6^A can be negatively regulated by microRNAs (miRNAs) (Cai et al., 2018). In eukaryotic cells, m^6^A methylation of messenger RNA (mRNA) is the most pervasive chemical modification, ahead of N^1^-methyladenosine (m^1^A), 5-methylcytosine (m^5^C), and N^7^-methylguanosine (m^7^G); it was first discovered in the 1970s (Desrosiers et al., 1974; Roundtree et al., 2017). The deposition of m^6^A is nearly identical in nascent and mature mRNA and is generally distributed in the exons of 3′ untranslated regions (UTRs) and stop codons (Ke et al., 2017; Shi et al., 2019) (Figure 1A). Recent studies have shown that some non-coding RNAs (ncRNAs) also act as coding RNAs, participating in peptide translation (Zhou et al., 2021). m^6^A modification has been detected in both coding and non-coding RNAs, including miRNAs, lncRNAs, and circular RNAs (circRNAs). As a prevalent post-transcriptional modification, it has a great impact on the fate of RNAs by binding with “m^6^A writers” and “m^6^A erasers” as well as recruiting “m^6^A readers” (Zaccara et al., 2019; Yi et al., 2020). Dynamic m^6^A methylation is vital for both normal biological processes and aberrant regulation in diseases (Li et al., 2017a; Du et al., 2017; Ma et al., 2017; Yang et al., 2017; Chen et al., 2018; Visvanathan et al., 2018; Han et al., 2019a; Wang et al., 2019a; Jia et al., 2019; Jin et al., 2019; Liu et al., 2019; Zhu et al., 2019; Wang et al., 2020a; Zhang et al., 2020a; Wang et al., 2020b; Guo et al., 2020; Hanniford et al., 2020; Liu et al., 2020; Wen et al., 2020; Zhang et al., 2021a; Huang et al., 2022) (Table 1).

**TABLE 1:** Roles of m^6^A modification in cancers.

Cancer type	m^6^A factors	Function	Alterations	Related RNA		Mechanisms	References
				Coding RNAs	Non-coding RNAs		
Glioblastomas (GBMs)	METTL3	Writer	Upregulated	SOX2 mRNA		METTL3 enhanced the stability of SOX2 mRNA which results in tumorigenesis and radioresistance of GBMs	Visvanathan et al. (2018)
	ALKBH5	Eraser	Upregulated		lncRNA FOXM1-AS	FOXM1-AS regulated a combination of ALKBH5 and FOXM1 transcripts to promote glioblastoma proliferation	Zhang et al. (2017)
Ocular Melanoma	YTHDF1	Reader	Downregulated	HINT2 mRNA		Reduction of YTHDF1 decreased translation of HINT2 mRNA to facilitate ocular melanoma	Jia et al. (2019)
Papillary Thyroid Cancer	FTO	Eraser	Downregulated	APOE mRNA		FTO decreased stability of APOE mRNA to attenuate growth of papillary thyroid cancer	Huang et al. (2022)
Breast Cancer	METTL3	Writer	Upregulated		miRNA let-7g	Inhibiting miRNA let-7g could upregulate METTL3 to promote proliferation of breast cancer	Cai et al. (2018)
Non-small-cell lung carcinoma (NSCLC)	METTL3	Writer	Upregulated		miR-33a	MiR-33a targeted 3′-UTR of METTL3 mRNA to insult NSCLC proliferation	Du et al. (2017)
	METTL3	Writer	Upregulated		lncRNA MALAT1	Assistant with YTHDF3, METTL3 could enhance stability of MALAT1 to sponge miR-1914-3p leading to NSCLC invasion and metastasis	Jin et al. (2019)
	YTHDF3	Reader	Upregulated		miR-1914-3p		
Liver Cancer	YTHDF2	Reader	Upregulated	OCT4 mRNA		YTHDF2 increased OCT4 expression to promote metastasis of liver cancer	Zhang et al. (2020a)
Hepatocellular Carcinoma (HCC)	METTL14	Writer	Downregulated		miR126	Downregulation of METTL14 restrains miR126 to facilitate HCC invasion and metastasis	Ma et al. (2017)
	METTL3	Writer	Upregulated	SOCS2 mRNA		METTL3 downregulates stability of to SOCS2 mRNA promote HCC progression	Chen et al. (2018)
	YTHDF2	Reader	Upregulated		miR-145	miR-145 could downregulate YTHDF2 to suppress proliferation of HCC cells	Yang et al. (2017)
Hepatoblastoma	METTL3	Writer	Upregulated	CTNNB1 mRNA		METTL3 increased expression of CTNNB1 to promote hepatoblastoma development	Liu et al. (2019)
Pancreatic Cancer (PC)	METTL14	Writer	Upregulated	PER1 mRNA		Elevated METTL4 promoted PER1 mRNA stability in YTHDF2-dependent manner to facilitate PC metastasis	Wang et al. (2020a)
	ALKBH5	Eraser	Downregulated	PER1 mRNA		ALKBH5 increased the expression of PER1 mRNA to suppress proliferation, migration and invasion and metastasis of PC.	Guo et al. (2020)
Esophageal Cancer (EC)	IGF2BP1	Reader	Upregulated	PEG1) mRNA		IGF2BP1 increased stability of PEG10 mRNA to promote proliferation and progression of EC.	Zhang et al. (2021a)
Ovarian Cancer (OC)	YTHDF1	Reader	Upregulated	EIF3C mRNA		YTHDF1 promoted translation of EIF3 to increase development and metastasis of OC.	Liu et al. (2020)
	ALKBH5	Reader	Upregulated	BCL-2 mRNA		ALKBH5 increased stability of BCL-2 mRNA to inhibit autophagy and promote tumorigenesis of OC.	Zhu et al. (2019)
Cervical Cancer (CC)	ALKBH5	Eraser	Upregulated		lncRNA GAS5	GAS5 could be stabilized by ALKBH5 and YTHDF2 to induce CC.	Wang et al. (2019a)
	YTHDF1	Reader	Upregulated	HK2 mRNA		YTHDF1 could stabilize HK2 mRNA methylated by METTL3 to develop CC.	Wang et al. (2020b)
Bladder Cancer	METTL3	Writer	Upregulated		miR221/222	METTL3 modulated the process of miR221/222 resulting in tumorigenesis of bladder cancer	Han et al. (2019a)
Prostate Cancer	CYCLINL1	Reader	Upregulated		NEAT1-1	CYCLINL1 was bound with non-coding RNA NEAT1-1 leading to metastasis of prostate cancer	Wen et al. (2020)
Melanoma	IGF2BP3	Reader			circRNA CDR1as	Interaction between CDR1as and IGF2BP3 was weakened by regulation of PRC2 to facilitate metastasis of melanoma	Hanniford et al. (2020)
Acute Myeloid Leukemia (AML)	METTL3	Writer	Upregulated	c-MYC, BCL2 and PTEN mRNAs		METTL3 accelerates c-MYC, BCL2 and PTEN mRNAs translation to develop AML.	Vu et al. (2017)
	FTO	Eraser	Upregulated	ASB2 and RARA mRNAs		FTO reduces the expression of ASB2 and RARA in leukemogenesis	Li et al. (2017a)

Methyltransferase-like 3 (METTL3), Methyltransferase-like 14 (METTL14), Wilms tumor 1-associated protein (WTAP), and auxiliary proteins form a molecular complex including vir-like m^6^A methyltransferase associated (VIRMA), RNA-binding motif protein 15 (RBM15 and RBM15B), Cbl proto-oncogene like 1 (CBLL1), and zinc finger CCCH domain-containing protein 13 (ZC3H13) (Wang et al., 2020c). These m^6^A methyltransferase can catalyze RNA methylation to insert a methyl substituent into the sixth N atom of adenosine in RNA. Using crystallization methods, it has been shown that METTL3 mainly has catalytic functions and METTL14 is a structural support factor; these loci function as m^6^A writers (Zeng et al., 2020). Another m^6^A methyltransferase, METTL16, could interact with mRNAs and small nuclear RNAs (snRNAs) using S-adenosylmethionine (SAM) as a methyl donor (Satterwhite and Mansfield, 2021). Fat mass and obesity-associated protein (FTO) and alkB homologue 5 (ALKBH5) are two common demethylases functioning as m^6^A erasers, reversibly removing m^6^A in nuclei (Jia et al., 2011; Zheng et al., 2013). FTO possesses multiple substrates including mRNA, tRNA, U RNAs, and RNAs transcribed from repetitive elements (Wei et al., 2018; Wei et al., 2022). m^6^A readers include YTHDF1, YTHDF2, YTHDF3, YTHDC1, and YTHDC2, which belong to the YT521-B homology (YTH) domain family and function downstream of m^6^A methylation or demethylation by m^6^A writers (Zhao et al., 2017). Insulin-like growth factor 2 mRNA-binding proteins (IGF2BP) have functions similar to those of m^6^A readers (Deng et al., 2018), mediating mRNA stability and translation (Figure 1B). The functions of these m^6^A readers mainly include specific binding to the m6A methylation region, weakening homologous binding to RNA-binding proteins, and altering the RNA secondary structure to alter protein-RNA interactions.

m^6^A is tightly associated with the post-transcriptional modification of gene expression by its deposition on RNA transcripts, thereby impacting tumorigenesis. ncRNAs also regulate the expression of m^6^A regulators, influencing the characteristics of cancers. Nevertheless, the relationship between m^6^A modification and GI cancer, especially the modification of ncRNAs, has not been summarized systematically. Here, we review the effects of m^6^A methylation on mRNAs and ncRNAs as well as the effects of ncRNAs on m^6^A regulators in GI cancer to explore the role of m6A modification in GI cancer, its potential as a diagnostic/prognostic marker, and its implications for therapy. Furthermore, we describe the prognostic and therapeutic value of m^6^A regulators in GI cancer.

## 2 m^6^A modification in GI cancer

Recently, aberrant levels of m^6^A and abnormal expression levels of m^6^A regulators have been found in GI cancer. These changes are mediated by various signaling pathways. Nevertheless, m^6^A regulators could be deposited on various oncogenes, or tumor suppressor genes, and these two factors might have opposite effects on tumorigenesis. The mechanisms underlying m^6^A modification and downstream effects are still unclear and have not been summarized systematically. Herein, we summarize the relationship between m^6^A and its associated coding and non-coding RNAs in GI cancer, to present a detailed overview of the contribution of epigenetic modifications in GI tumors.

### 2.1 Relationship between m^6^A and coding RNAs in gastrointestinal cancer

Methyltransferase, demethylase, and recognition factors play important roles in the molecular mechanisms of action of m^6^A RNA methylation, modulating mRNA stability, splicing, nuclear export, and translation (Table 2).

**TABLE 2:** m^6^A modification and mRNA in gastrointestinal cancers.

m^6^A modification on mRNA in gastrointestinal cancers						
m^6^A regulator		Cancer type	Targeted molecular	Mechanism	Related m^6^A reader	References
Writers	METTL3	Esophageal squamous cell carcinoma (ESCC)	NOTCH1 mRNA	Increase RNA stability		Han et al. (2021)
			APC mRNA	Enhance degradation	YTHDF	Wang et al. (2021a)
			EGR1 mRNA	Increase RNA stability	YTHDF	Li et al. (2021a)
		Gastric Cancer(GC)	HDGF mRNA	Increase RNA stability	IGF2BP3	Wang et al. (2020d)
			ZMYM1 mRNA	Increase RNA stability		Yue et al. (2019)
			BATF2 mRNA	Decrease RNA stability		Xie et al. (2020)
			PBX1 mRNA	Increase RNA stability		Liu et al. (2022a)
			MYC mRNA	Promote translation		Yang et al. (2020a)
			SPHK mRNA	Promote translation	YTHDF1	Huo et al. (2021)
		Colorectal Cancer(CRC)	CCNE1 mRNA	Increase RNA stability		Zhu et al. (2020a)
			SEC62 mRNA	Increase RNA stability	IGF2BP2	He et al. (2019b), Liu et al. (2021)
			PLAU mRNA	Increase RNA stability		Yu et al. (2022)
			HK2 and GLUT1 mRNA	Increase RNA stability		Shen et al. (2020), Wang et al. (2021a)
			CRB3 mRNA	Decrease RNA stability		Yang et al. (2021a)
			SOX2 mRNA	Increase RNA stability	IGF2BP2	Huang et al. (2018), Li et al. (2019)
			K2F26B mRNA	Enhance degradation	YTHDF2	Huang et al. (2018)
	METTL14	Colorectal Cancer(CRC)	SOX4 mRNA	Decrease RNA stability	YTHDF2	Chen et al. (2020a)
			ARRDC4 mRNA	Decrease RNA stability	YTHDF2	Wang et al. (2021b)
			KLF4 mRNA	Increase RNA stability	IGF2BP2	Wang et al. (2021c)
	METTL16	Gastric Cancer(GC)	Cyclin D1 mRNA	Decrease RNA stability		Huo et al. (2021)
Erasers	FTO	Gastric Cancer(GC)	HOXB13 mRNA	Promote expression		Chen et al. (2020a)
			caveolin-1 mRNA	Enhance degradation		Wang et al. (2021b)
			MYC mRNA	Increase RNA stability		Warda et al. (2017)
		Colorectal Cancer(CRC)	MYC mRNA	Promote expression		Wang et al. (2021d)
	ALKBH5	Gastric Intestinal Metaplasia (IM)	ZNF333 mRNA	Increase RNA stability	YTHDF2	Ge et al. (2020)
		Gastric Cancer(GC)	PKMYT1 mRNA	Decrease RNA stability	IGF2BP3	Yang et al. (2021b)
Readers	YTHDC2	Gastric Cancer(GC)	YAP mRNA	Promote translation		Yue et al. (2020)
	YTHDF1	Gastric Cancer(GC)	FZD7 mRNA	Promote translation		Zhang et al. (2021b)
		Colorectal Cancer(CRC)	ARHGEF2 mRNA	Promote translation		Xiao et al. (2021)
	IGF2BP2	Colorectal Cancer(CRC)	YAP mRNA	Promote translation		Yue et al. (2021a)
	IGF2BP3	Colorectal Cancer(CRC)	CCD1 mRNA	Increase RNA stability		Hu et al. (2022)
			VEGF mRNA			
**m** ^**6**^ **A modification on mRNA in gastrointestinal cancers**						
**m** ^**6**^ **A regulator**		**Cancer type**	**Upstream regulatory molecular**	**Alterations**	**Role in cancer**	**References**
Writers	METTL3	Gastric Cancer(GC)	HBXIP	Upregulation	Oncogene	Shen et al. (2020)
	WTAP	Colon Cancer	TTC22	--	Oncogene	Wang et al. (2022a)

#### 2.1.1 m^6^A modification on coding RNAs

##### 2.1.1.1 Writers of m^6^A

###### 2.1.1.1.1 METTL3

METTL3 is an m^6^A methyltransferase that has been studied extensively owing to its roles in various cancers. For example, METTL3 mediates carcinogenesis in GBM by influencing characteristics of mRNA, and its m^6^A modification has been studied in AML as much as in GBM (He et al., 2019a). METTL3 contributes substantially to GI cancer, and the impact of its dysregulation on targeted mRNAs leads to different outcomes.

Many studies have demonstrated that METTL3 could affect the stabilization of mRNA. For example, METTL3 promotes the development of esophageal squamous cell carcinoma (ESCC) through the stabilization of NOTCH1 and EGR1 mRNA, followed by activation of Notch and EGR1/Snail signaling pathways (Li et al., 2021a; Han et al., 2021). The activation of *METTL3* transcription promotes the m^6^A methylation of Hepatoma-Derived Growth Factor (*HDGF*) mRNA, and subsequent binding to IGF2BP3, an m^6^A reader, leading to increased stability of *HDGF* mRNA in GC (Wang et al., 2020d). Yue et al. reported that in GC, METTL3 targets zinc finger MYM-type containing 1 (*ZMYM1*), increasing its expression, and thereby influencing the process of EMT (Yue et al., 2019). The mRNA levels of the basic leucine zipper ATF-like transcription factor 2 (*BATF2*) decrease in response to increased METTL3 in GC, resulting in a decrease in the stability of *BATF2* mRNA (Xie et al., 2020). In addition to the elevated level of METTL3 in GC, the locus is correlated with lung/lymph node metastasis, due to its stabilizing of Pre-B-cell leukemia homeobox 1 (*PBX1*), an oncogene (Liu et al., 2022a). Zhu et al. found that METTL3 could lead to stabilization of *CCNE1* mRNA by binding to its 3′-UTR and methylating it, promoting CRC (Zhu et al., 2020a). Similarly, some studies have shown that cancer-related oncogene can be modified by METTL3 to enhance their mRNA stability in CRC (Liu et al., 2021; Yu et al., 2022). Furthermore, METTL3 is involved in stabilizing the mRNA of *HK2* and *SLC2A1* (*GLUT1*), and degrading the mRNA of *APC;* all these effects are associated with the glycolysis and proliferation of tumor cells (Shen et al., 2020; Wang et al., 2021a). A recent study has shown that knockdown of METTL3 could prevent the degradation of *CRB3* mRNA in CRC, mediating the activation of the Hippo signaling pathway (Yang et al., 2021a). Li et al. also reported that increased METTL3 expression in CRC is associated with metastasis; furthermore, METTL3 can reduce the degradation of the downstream factor SRY (sex determining region Y)-box 2 (*SOX2*), by methylating coding sequence regions by an IGF2BP2-dependent mechanism, in which K homology domains of IGF2BPs are responsible for tumorigenesis (Huang et al., 2018; Li et al., 2019). Accordingly, METTL3 is a candidate target for the treatment of CRC and other cancers.

In addition to stabilization, METTL3 could influence translation. Altered METTL3 is important for GC development; however, the regulatory processes downstream of m^6^A factors are still unclear. METTL3 is upregulated, increasing the translation of the oncogene *MYC* in GC and promoting proliferation (Yang et al., 2020a). METTL3 and YTHDF1 positively regulate the translation of Sphingosine kinase (*SPHK*), leading to the migration and invasion of GC (Huo et al., 2021). However, given that METTL3 could facilitate cancer progression through an anchoring effect in a non-m^6^A modification manner, there are still unknown mechanisms in the METTL3 regulatory role in GI carcinogenesis. This suggests that METTL3 may promote the translation of certain epigenetic factors in the cytoplasm in a m^6^A-independent manner.

###### 2.1.1.1.2 METTL14

As a homolog of METTL3, METTL14 is also aberrantly expressed in tumorigenesis. METTL14 acts as an anti-oncogene in CRC, abolishing *SOX4* mRNA stability and facilitating *SOX4* mRNA degradation in a YTHDF2-dependent manner, preventing metastasis in CRC (Chen et al., 2020a). Moreover, arrestin domain-containing 4 (*ARRDC4*), another target of METTL14, could be degraded via m^6^A modification by METTL14 and YTHDF2, resulting in low expression of the EMT regulator ZEB1 (Wang et al., 2021b). The association between colorectal anti-cancer gene *KLF4* and metastasis is inhibited by reduced METLL14, which promotes *KLF4* mRNA degradation in a IGF2BP2-dependent manner in CRC (Wang et al., 2021c). The role of METTL14 in tumor development is not limited to its effect on mRNA stability; it also affects ncRNAs such as circRNAs. This is detailed in the following section.

###### 2.1.1.1.3 METTL16

Similar to METTL3 and METTL14, another m^6^A methyltransferase, METTL16, has been found to be involved in the processing of pre-mRNA by interacting with the methylation of U6 snRNA, where it can bind to the 5′ splice sites of pre-mRNA (Warda et al., 2017). The downregulation of another methyltransferase, METTL16, inhibits the proliferation of GC cells by suppressing the GC cell cycle in G1/S phase and decreasing cyclin D1 mRNA stability (Wang et al., 2021d). As the most studied m^6^A regulators, “writers” can be viewed as novel targets for facilitating the treatment of GI cancer.

##### 2.1.1.2 Erasers of m^6^A

###### 2.1.1.2.1 FTO

FTO catalyzes the oxidative demethylation of m^6^A-modified nuclear RNA (Jia et al., 2011). A recent study has revealed that FTO acts as an oncogene, demethylating the mRNA of the Homeobox gene *HOXB13*, which augments the expression of HOXB13 in GC (Guo et al., 2021a). Other studies have suggested that the upregulation of FTO in GC, especially in cases with liver metastasis, promotes the degradation of caveolin-1 mRNA by reducing m^6^A deposition, impeding mitochondrial fission and inducing GC metastasis (Zhou et al., 2022). Nevertheless, the level of FTO was downregulated in the peripheral blood of patients with GC (Ge et al., 2020), contrary to its expression pattern in GC tissues. A clinical trial has revealed that FTO can demethylate *MYC* mRNA, sustaining its stability in GC cells and mediating proliferation, migration, and invasion of GC (Yang et al., 2021b). However, METTL3 and FTO have opposite functions in GC; still, their effects on MYC expression have been shown to be similar. The upregulation of FTO could also regulate MYC expression in CRC via the miR-96/AMPKα2/FTO/m6A/MYC axis (Yue et al., 2020). Subsequently, Zhang et al. reported that FTO facilitates CRC proliferation by targeting the MAF1/c-MYC axis, which can be inhibited by glycogen synthase kinase 3 beta (GSK3β) (Zhang et al., 2021b).

###### 2.1.1.2.2 ALKBH5

ALKBH5 functions in esophageal cancer cells (ESCC), and its overexpression results in the inhibition of proliferation, migration, and invasion by arresting cells in the G1 phase (Xiao et al., 2021); however, demethylated mRNA of *ALKBH5* and related signaling pathways in ESCC are unclear. The demethylation of ZNF333 by ALKBH5 leads to a reduction in the degradation of *ZNF333* mRNA, and this is dependent on YTHDF2 recognition, and hyperactivation of NF-κB to induce gastric intestinal metaplasia (IM) (Yue et al., 2021a). *In vitro* and *in vivo* assays revealed that the downregulation of ALKBH5 can increase the expression level of PKMYT1 by maintaining its stability with the assistance of IGF2BP3 via the demethylation of PKMYT1 in GC (Hu et al., 2022). Considering that m^6^A modification is a reversible process, “erasers” play an important role in carcinogenesis and tumor progression.

##### 2.1.1.3 Readers of m^6^A

###### 2.1.1.3.1 YT521-B homologues

Most readers of m^6^A mediate the fates of RNA following modification by m^6^A writers. In processes related to tumorigenesis, m^6^A readers may lead to aberrant changes in targeted RNAs via variation in m^6^A readers themselves or misinterpretation of m^6^A writers. For example, YTHDC2, the first studied m^6^A reader, can recognize m^6^A sites on *YAP* mRNA, enhancing its translation (rather than influencing its mRNA level) and promoting proliferation, invasion, and metastasis of GC (Yuan et al., 2022). ALKBH5 levels can also increase due to the upregulation of YAP, forming a positive feedback loop. YTHDF1 acts as an oncogene, promoting GC progression and metastasis by recognizing frizzled 7 (*FZD7*) mRNA based on m^6^A via the activation of the Wnt/FZD7/β-catenin pathway (Pi et al., 2021). It has recently been demonstrated that YTHDF1 is highly expressed in CRC and enhances the translation of its target, *ARHGEF2,* via RhoA signaling (Wang et al., 2022a).

###### 2.1.1.3.2 Insulin-like growth factor 2 mRNA-binding proteins

IGF2BP1/2/3 proteins are newly identified m^6^A readers able to recognize m^6^A modifications and promote stability (Huang et al., 2018). Elevated IGF2BP2 has been detected in CRC, and its function has been found to be the same as that of YTHDC2 in GC, i.e., it promotes the stability and translation of YAP by recognizing its mRNA, activating ErbB2 and leading to a malignant phenotype in CRC cells (Cui et al., 2021a). Compared with the levels in normal colon tissues, IGF2BP3 levels are elevated in CRC (Yang et al., 2020b). Knockdown of IGF2BP3 results in decreased efficacy of reading the m^6^A sites of the mRNAs of the cell cycle protein Cyclin D1 (*CCND1*) and Vascular endothelial growth factor (*VEGF*), impairing their mRNA stability, inhibiting proliferation, and repressing angiogenesis (Yang et al., 2020b). Recently, studies of m^6^A readers have not been limited to their downregulated targets, but also include their up-regulatory mechanisms. The functions of m^6^A readers need further specific explorations to be applied in GI cancer intervention.

#### 2.1.2 Factors upstream of m^6^A regulators

Some studies have focused on the upstream regulatory mechanism of m^6^A regulators in GI cancer, and have found that some coding RNAs can influence the level of m^6^A. METTL3 has been confirmed to be a downstream target of specific signaling pathways, and to play a critical role in EMT progression, by elevating m^6^A levels (Song and Zhou, 2021) (Table 2). As it functions in breast cancer, hepatitis B X-interacting protein (HBXIP) could positively regulate METTL3 levels in GC cells, where METTL3 could interact with the oncogene *MYC* to increase m^6^A deposition, promoting proliferation, invasion, and metastasis (Yang et al., 2020a). Chen proposed a more complex mechanism of m^6^A regulation in CRC involving gut microbiota, in which *Fusobacterium nucleatum* facilitates metastasis by downregulating METTL3 via the inhibition of the HIPPO signaling pathway, activation of YAP signaling pathways, and elevation of the expression of the oncogene *KIF26B* due to reduced YTHDF2-dependent degradation (Chen et al., 2022a; You et al., 2022). These results indicate that the up-regulatory factors of m^6^A modification need further exploration to understand what influences the m^6^A methylation.

### 2.2 Relationship between m^6^A and non-coding RNA in gastrointestinal cancer

#### 2.2.1 m^6^A modifications on ncRNAs

Increasing evidence suggests that ncRNAs are involved in biological processes and disease development. They function by regulating gene expression but lack protein-coding capacity. ncRNAs can be divided into small ncRNAs and long ncRNAs using a threshold length of 200 nucleotides (Beermann et al., 2016). As ncRNAs are related to carcinogenesis, the m^6^A-mediated regulation of gene expression and interactions with related ncRNAs contribute to pathological processes (Chen et al., 2020b). For example, the m^6^A eraser ALKBH5 is colocated with the lncRNA NEAT1 in the nuclei of GC cells, leading to the demethylation of NEAT1, thereby influencing invasion and metastasis (Zhang et al., 2019a) (Table 3).

**TABLE 3:** m^6^A modification on ncRNAs of gastrointestinal cancers.

Cancer type	Year	m^6^A regulator		Alterations	Role in cancer	Related ncRNAs	Function and pathways	References
Esophageal cancer (EC)	2021	Eraser	FTO	Upregulation	Oncogene	LINC00022	De-stabilizing p21 protein by its ubiquitination	Cui et al. (2021b)
	2021	Eraser	ALKBH5	Downregulation	Anti-oncogene	miR-194-2	Suppressing YAP/TAZ nuclear translocation to enhance transcription of Hippo pathway upstream genes	Chen et al. (2021a)
	2021	Reader	HNRNPA2B1	Upregulation	Oncogene	miR-17-92	--	Li et al. (2021b)
	2021	Reader	YTHDC1	--	--	lncRNA MALAT1	Remodeling the composition of nuclear speckles	Wang et al. (2021e)
Gastric cancer(GC)	2019	Eraser	ALKBH5	Upregulation	Oncogene	lncRNA NEAT1	Regulating the expression of downstream genes of EZH2	Zhang et al. (2019a)
	2020	Writer	METTL3	Upregulation	Oncogene	miR-17-92	Activating AKT/mTOR pathway	Sun et al. (2020)
	2022	Writer	METTL3	Upregulation	Oncogene	lncRNA THAP7-AS1	Activating PI3K/AKT pathway	Liu et al. (2022b)
	2022	Writer	METTL14	Downregulation	Anti-oncogene	miR-30c-2-3p, circORC5	Upregulation of miR-30c-2-3p and downregulation of AKT1S1 and EIF4B	Fan et al. (2022)
Colorectal cancer (CRC)	2016	Reader	IGF2BP2	Upregulation	Oncogene	miR-195	Regulating the expression of downstream genes of RAF1	Chen et al. (2022b)
	2019	Writer	METTL3	Upregulation	Oncogene	miR-1246	Activating RAF/MEK/ERK pathway	Peng et al. (2019)
	2019	Writer	METTL3	Upregulation	Oncogene	lncRNA RP11	Proteasomal degradation of Zeb1 prevented by RP11/hnRNPA2B1/mRNA complex	Wu et al. (2019)
	2019	Reader	YTHDC1 IGF2BP2	Upregulation	Oncogene	circNSUN2	Forming a circNSUN2/IGF2BP2/HMGA2 RNA-protein ternary complex in the cytoplasm	Chen et al. (2019a)
	2020	Writer	METTL14	Downregulation	Anti-oncogene	lncRNA XIST	Activating m6A-YTHDF2 dependent pathway	Yang et al. (2020c)
	2021	Writer	METTL3	Upregulation	Oncogene	lncRNA PTTG3P	Activating Hippo pathway	Zheng et al. (2021)
	2021	Writer	METTL3	Upregulation	Oncogene	circ1662	Promoting nuclear transport of YAP1 and inhibiting the expression of Smad3	Chen et al. (2021b)
	2022	Writer	METTL3	Upregulation	Oncogene	miR-181d-5p	Targeting NCALD to regulate the sensitivity to 5-FU.	Pan et al. (2022)
	2022	Reader	YTHDF2	Upregulation	Oncogene	circ_0003215	Suppressing the pentose phosphate pathway	Chen et al. (2022b)

##### 2.2.1.1 Esophageal cancer

Recently, the deregulation of m^6^A factors has been found to play a key role in the occurrence of esophageal cancer (EC). Genetic mutation of m^6^A regulators has been demonstrated to be associated with ESCC patients’ prognosis (Guo et al., 2021b; Zhao et al., 2021). ESCC-associated m^6^A eraser FTO assists in promoting cell-cycle progression and proliferation by decreasing the m^6^A level of the LINC00022 transcripts, upregulating the expression of LINC00022 (Cui et al., 2021b). Similar regulatory function is also observed in the relationship between m^6^A reader HNRNPA2B1and miR-17-92 in ESCC (Li et al., 2021b). MALAT1-m^6^A recognition by YTHDC1 has been implicated in the metastasis of EC as demonstrated *in vitro* and *in vivo* (Wang et al., 2021e). As for mechanism, one study has shown that ALKBH5 can demethylate pri-miR-194-2, decreasing the level of miR-194-2, which functions as inhibitor in ESCC (Chen et al., 2021a). Current studies mostly address the phenotypic regulation of tumors by m^6^A factors, but specific modification mechanisms also require further study.

##### 2.2.1.2 Gastric cancer

**FIGURE 2:**
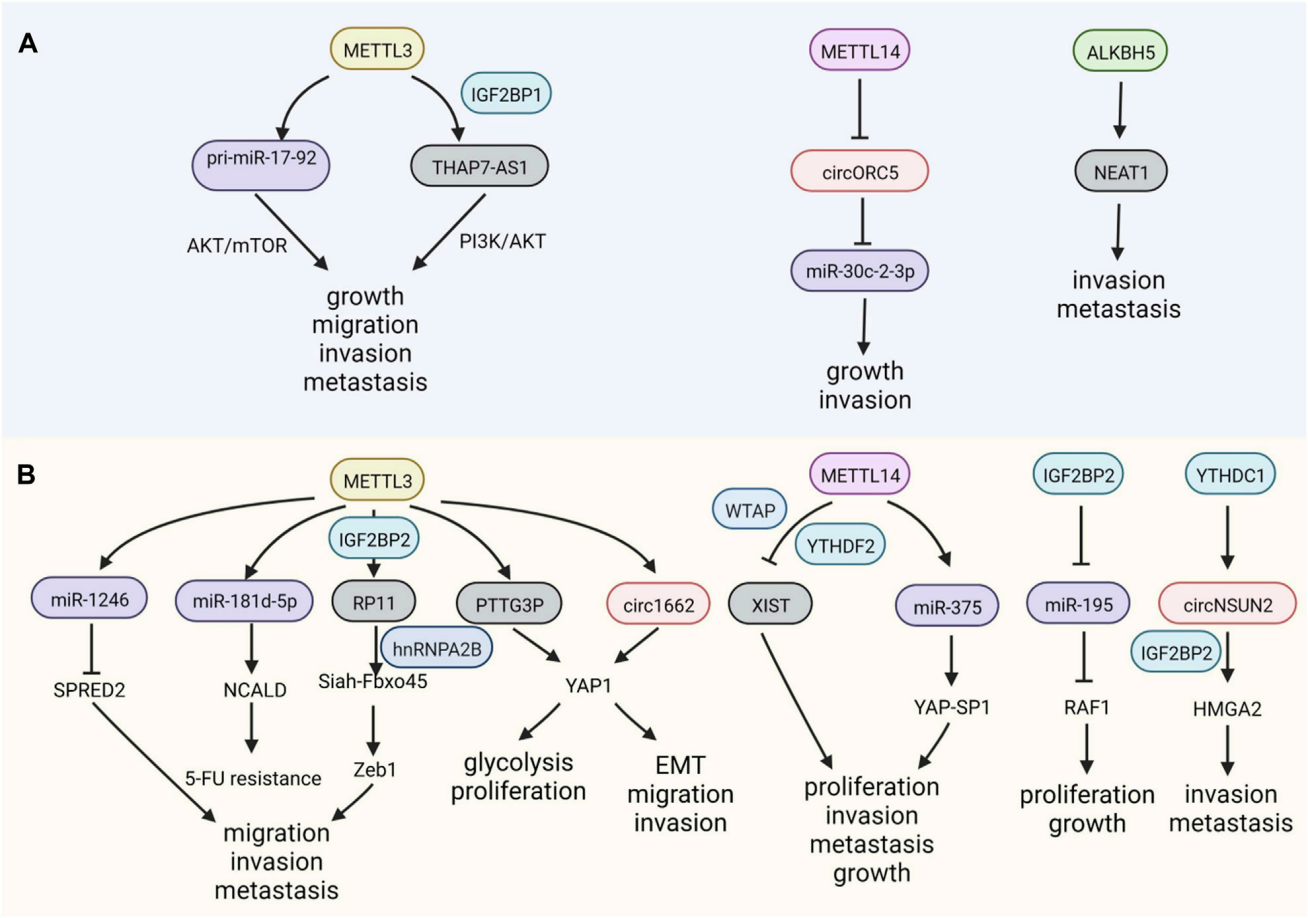
m^6^A modifications on non-coding RNAs (ncRNAs) in GI cancers. **(A)** m^6^A regulators have positive or negative functions on ncRNAs in gastric cancer (GC). **(B)** m^6^A modulates the expression of ncRNAs, influencing colorectal cancer (CRC).

M^6^A modification can mediate GC development by controlling the process and fate of ncRNAs, including their maturation, stability, and transportation. For example, the overexpression of METTL3 facilitates the maturation of pri-miR-17-92, which targets AKT/mTOR pathway, promoting GC growth and peritoneal metastasis (Sun et al., 2020). The lncRNA THAP7-AS1 is a downstream target of METTL3 and its effects are dependent on IGF2BP1, whose stability is maintained and expression is increased, exerting carcinogenic effects in GC (Liu et al., 2022b). METTL14, a writer of m^6^A, and METTL3 have opposite regulatory effects in GC. The depletion of METTL14 facilitates the growth of GC and abolishes m^6^A on circORC5, augmenting its expression by sponging miR-30c-2-3p (Fan et al., 2022) (Figure 2A).

##### 2.2.1.3 Colorectal cancer

The upregulation of METTL3, an m^6^A writer, is also critical for the migration and invasion of CRC via its effects on miR-1246, whose downstream targets include the anti-oncogene *SPRED2*, which functions through the Raf/MEK/ERK pathway (Peng et al., 2019). In addition to the malignant characteristics of CRC, METTL3 can also regulate chemotherapeutic resistance by processing miR-181d-5p via DiGeorge Syndrome Critical Region 8 (DGCR8) (Pan et al., 2022). The invasion and metastasis of CRC can also be modulated by the lncRNA RP11 methylation mediated by METTL3, and the formation of the RP11/hnRNP2B1/mRNA complex leads to the upregulation of the EMT-related factor Zeb1 (Wu et al., 2019). METTL3 helps stabilize the lncRNA PTTG3P, which is recognized by IGF2BP2, thereby promoting CRC (Zheng et al., 2021). In addition to miRNAs and lncRNAs modified by m^6^A, recent studies have also focused on the m^6^A methylation of circRNAs. Interestingly, binding to circ1662 flanking reverse repeats, METTTL3 upregulates circ1662, facilitating YAP1 nuclear transportation, which inhibits the EMT-related gene *SMAD3* and promotes migration and invasion in CRC (Chen et al., 2021b).

The lncRNA X inactivate-specific transcript (XIST) is methylated by METTL3, WTAP, RBM15, and RBM15B, and is recognized by YTHDC1, inactivating gene transcription on the X chromosome (Patil et al., 2016). The knockdown of METTL14 contributes to the progression of CRC via the recognition of YTHDF2, which modifies XIST, which is related to tumorigenesis in CRC by reducing its degradation (Patil et al., 2016; Yang et al., 2020c), similar to the function of METTL14 in GC.

YTH domain-containing protein 1 (YTHDC1) is bound to RNA in nuclei, where it impacts RNA splicing. Dependent on the m^6^A reader YTHDC1, circNSUN2 could be exported to the cytoplasm to combine with another reader, IGF2BP2; the resulting complex promotes the stability of high mobility group AT-hook 2 (*HMGA2*) mRNA, facilitating the metastasis of CRC (Chen et al., 2019a). Elevated IGF2BP2 expression maintains the stability of *RAF1* mRNA by reversing miR-195-mediated degradation, promoting CRC (Ye et al., 2016). Similarly, YTHDF2 can inhibit circ_0003215 expression by degrading its RNA, leading to metabolic reprogramming of CRC cells (Chen et al., 2022b). These reports reveal that several special m^6^A regulators are responsible for ncRNA processing in GI cancer (Figure 2B).

#### 2.2.2 ncRNAs impact the expression and function of m6A-regulating proteins

ncRNAs have both positive and negative regulatory roles in m^6^A modification in GI cancer (Table 4). For example, miR-186 has been validated to suppress the expression of HNRNPC, a little-studied m^6^A regulator, facilitating migration and invasion in ESCC, whose regulatory mechanism still remains unclear (Li et al., 2021b). Furthermore, the repression of miR455-3p can increase the m^6^A modification of Heat shock transcription factor (*HSF1*) mRNA by competing with METTL3 to promote CRC (Song et al., 2020). Another miRNA, miR-96, contributes substantially to CRC development by inhibiting FTO, elevating m^6^A modification (Yue et al., 2020). The lncRNA LINC00470 is highly upregulated in GC in a manner dependent on the m^6^A binding proteins METTL3 and YTHDF2, exerting structural effects on *PTEN* mRNA, leading to its instability and degradation and promoting GC (Yan et al., 2020). The lincRNA NRON is overexpressed in GC, recruiting the m^6^A eraser ALKBH5 and decreasing the decay of Nanog transcripts by reducing m^6^A levels on Nanog mRNA (Wang et al., 2021f). MiR-1269b suppresses GC migration and invasion by targeting METTL3 (Kang et al., 2021). The lncRNA BLACAT2 promotes GC development via miR-193b-5p/METTL3 by obstructing apoptosis (Hu et al., 2021). Similarly, the lncRNA LINC000240 acting as a sponge facilitates the malignant phenotype of GC via the miR-338-5p/METTL3 axis (Wang et al., 2021b). In colon cancer, suppression of the lncRNA HOTAIR was found to downregulate the expression of IGF2BP2; HOTAIR can inhibit EMT, proliferation, cell cycle, metastasis, and invasion and facilitate cell apoptosis (Wu et al., 2018). Therefore, ncRNAs are important for m^6^A modulation because they control the expression of m^6^A.

**TABLE 4:** ncRNAs modification on m^6^A of gastrointestinal cancers.

Cancer type	Year	ncRNAs	Alterations	Role in cancer	Related m^6^A regulator	References
Esophageal squamous cell carcinoma (ESCC)	2021	miR-186	Downregulation	Anti-oncogene	HNRNPC	Li et al. (2021b)
Gastric cancer (GC)	2017	miR-34a	Downregulation	Anti-oncogene	IGF2BP3	Zhou et al. (2017)
	2019	miR-4429	Downregulation	Anti-oncogene	METTL3、IGF2BP2	He et al. (2019b)
	2020	LINC00470	Upregulation	Oncogene	METTL3、YTHDF2	Yan et al. (2020)
	2021	miR-1269b	Downregulation	Anti-oncogene	METTL3	Kang et al. (2021)
	2021	miR-338-5p	Downregulation	Anti-oncogene	METTL3	Zhang et al. (2021c), Wang et al. (2021g)
	2021	lncRNA BLACAT2	Upregulation	Oncogene	METTL3	Xiao et al. (2019)
	2021	lncRNA LINC000240	Upregulation	Oncogene	METTL3	Patil et al. (2016)
	2021	LncRNA NRON	Upregulation	Oncogene	ALKBH5	Wang et al. (2021f)
	2022	lncRNA LINC02253	Upregulation	Oncogene	METTL3	Gao et al. (2022)
	2022	circRPMS1	Upregulation	Oncogene	METTL3	Zhang et al. (2022)
Colorectal cancer (CRC)	2018	lncRNA HOTAIR	Upregulation	Oncogene	IGF2BP2	Wu et al. (2018)
	2019	LINRIS	Upregulation	Oncogene	IGF2BP2	Wang et al. (2019b)
	2019	LncRNA GAS5	Downregulation	Anti-oncogene	YTHDF3	Ni et al. (2019)
	2020	miR455-3p	Downregulation	Anti-oncogene	METTL3	Song et al. (2020)
	2020	miR-96	Upregulation	Oncogene	FTO	Yue et al. (2020)
	2020	LINC00266-1	Upregulation	Oncogene	IGF2BP1	Zhu et al. (2020b)
	2021	miR-6165	Downregulation	Anti-oncogene	YTHDF2	Li et al. (2021d)
	2021	LINC01605	Upregulation	Oncogene	METTL3	Yue et al. (2021b)
	2021	LINC00460	Upregulation	Oncogene	IGF2BP2	Hou et al. (2021)
	2022	circLPAR1	Downregulation	Anti-oncogene	METTL3	Zheng et al. (2022)

##### 2.2.2.1 Gastric cancer

**FIGURE 3:**
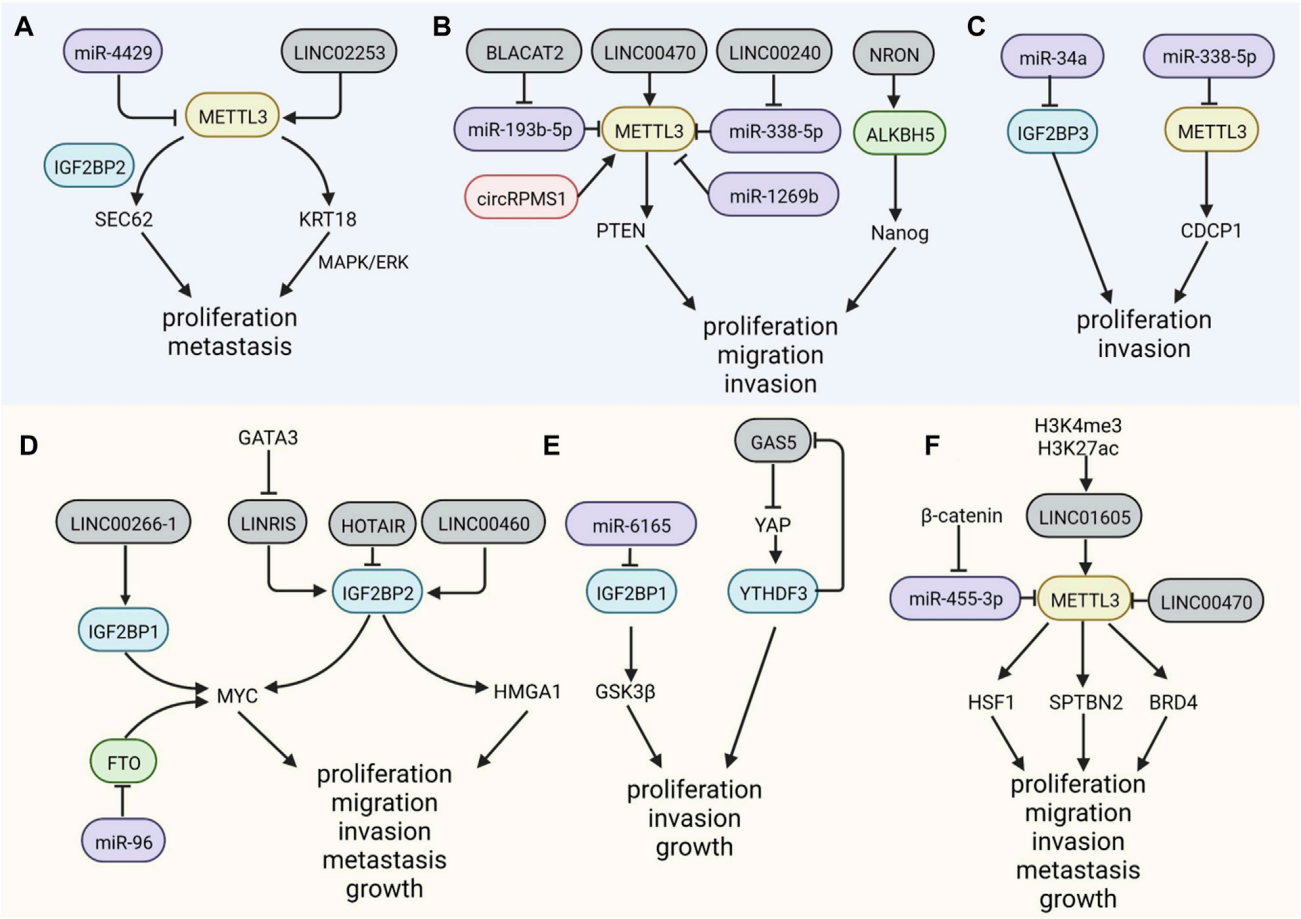
Non-coding RNAs participate in the regulation of m^6^A factors. **(A–C)**. MicroRNAs, lincRNAs and circRNA can modulate the expression level of m^6^A regulators mediating the development of GC. **(D–F)**. NcRNAs also modify the methyl group m^6^A regulators in CRC.

As the most widely known m^6^A writer, METTL3 is upregulated in distinct cancer types. In GC, *SEC62*, a gene involved in carcinogenesis, is methylated by METTL3 (Liu et al., 2021), recruiting the m^6^A reader IGF2BP2 and stabilizing *SEC62* mRNA; however, this carcinogenic effect can be attenuated by miR-4429 (He et al., 2019b). MiR-338-5p is downregulated by embryonic ectoderm development protein (EED) and then reduces METTL3 inhibition and increases the translation of CUB domain containing protein 1 (CDCP1), inducing proliferation and invasion in GC (Zhang et al., 2021c). Gao et al. reported that the lncRNA LINC02253 stabilizes *KRT18* mRNA by recruiting METTL3, without affecting its expression, thus promoting GC proliferation and metastasis (Gao et al., 2022). In addition, IGF2BP3 plays a significant role in GC propagation and metastasis, and its effects are facilitated by miR-34a silencing (Zhou et al., 2017). CircRNAs are a well-studied group of ncRNAs with a covalently closed structure; they function as sponges, assimilating miRNAs and proteins, and have a vital role in the development of many diseases. A recent study has clarified the function of circRPMS1 in Epstein-Barr virus-associated gastric carcinoma (EBVaGC) and suggested that via the interaction with Sam68, circRPMS1 activates *METTL3* (Zhang et al., 2022) (Figure 3A).

##### 2.2.2.2 Colorectal cancer

In CRC, METTL3 could interact with LINC1605 in the cytoplasm, regulating the translation of downstream factors and inducing malignant characteristics (Yue et al., 2021b). The expression of m^6^A reader YTHDF2 is inhibited by miR-6165, by binding to the 3′UTR of *YTHDF2* mRNA, leading to stabilization of m^6^A transcripts of *GSK3β*, downstream of YTHDF2, and inactivation of the Wnt/β-catenin/Cyclin D1 pathway, suppressing CRC carcinogenesis (Li et al., 2021d). Large intergenic non-coding RNAs (lincRNAs) are found to be modified by m^6^A, and most common motifs are GG/A(m^6^A)CH, different from the motifs in mRNAs (Xiao et al., 2019); m^6^A could be regulated by lincRNAs. IGF2BP2s, functioning as m^6^A readers, are highly blocked by the downregulation of LINRIS, a kind of lincRNA, via the ubiquitination-autophagy pathway, destroying its stability and the autophagy-lysosome pathway and assisting its degradation in CRC (Wang et al., 2019b). Long intergenic non-coding RNA 460 (LINC00460) is a novel non-coding RNA; its overexpression is associated with the progression of CRC. LINC00460 can increase the m^6^A modification of high mobility group A1 (*HMGA1*) mRNA by binding to IGF2BP2s and ATP-dependent RNA helicase A (DHX9), which enhances the stability of HMGA1 (Hou et al., 2021). Another lncRNA, GAS5, impedes the progression of CRC via the phosphorylation and degradation of YAP by the negative regulation of the m^6^A reader YTHDF3 (Ni et al., 2019). Some lncRNAs have the ability to encode proteins involved in m^6^A regulation. A peptide encoded by LINC00266-1, called “RNA binding regulatory peptide” (RBRP), can interact with IGF2BP1 and the complex targets c-Myc to facilitate its stability, promoting CRC (Zhu et al., 2020b). *In vitro* and *in vivo* assays have shown that circLPAR1 in exosomes can sponge RNA-binding proteins (RBP) elF3h, reducing their binding to METTL3 in CRC cells, resulting in decreased translation of *BRD4* mRNA and the suppression of CRC (Zheng et al., 2022) (Figure 3B). Overall, the evidence of the ncRNAs regulatory function on m^6^A, by coding or binding with m^6^A regulators, provides novel targets. The pathways involved in this relationship, such as cancer metabolism, remain limited, and related ncRNAs might be regulators that inhibit malignant phenotype in GI cancer.

## 3 Clinical value of m^6^A modification in GI cancer

### 3.1 Prognostic biomarkers

The identification of m^6^A-associated protein-coding genes in pan-cancer analyses has provided novel candidate targets for clinical diagnosis and treatment (Shen et al., 2021). Recent evidence suggests that m^6^A-associated ncRNAs could be used to construct prediction models for prognosis in GI cancer (Wang et al., 2022b; Xu et al., 2022). Due to dynamic and reversible changes in m^6^A regulators during cancer development, m^6^A methylation could serve as a prognostic biomarker to guide therapeutic schemes for numerous cancer types (Ji G et al., 2020; Jin et al., 2021). The main enzyme of m^6^A modification, METTL3, could facilitate tumor progression by depositing m^6^A modification on key transcripts. Furthermore, tumor progression may be independent of the catalytic activity of METTL3, but be related to recruitment of eukaryotic translation initiation factors into the translation initiation complex. METTL3 is upregulated in both GC and CRC, and the elevation in METTL3 is a prognostic factor for poor overall survival (OS) and disease-free survival (DFS) (Li et al., 2019; Yue et al., 2019; Wang et al., 2020d). Furthermore, METTL3 and IGF2BP3 have been reported to be independent factors for ESCC prognosis (Guo et al., 2021b). In addition, low level of METTL14 is related to a poor prognosis in GC and CRC patients, and to the opposite in ESCC patients (Chen et al., 2020b; Xu et al., 2020; Fan et al., 2022). It can be seen that METTL14 plays a suppressive role in GI cancer by targeting key downstream molecules. The m^6^A erasers FTO and ALKBH5 are also associated with a worse OS (Liu et al., 2022a; Zhou et al., 2022), which could modulate the metabolism of cancer cells and facilitate immune escape. Similarly, the upregulation of the m^6^A reader IGF2BP3 has been identified as a new biomarker of many cancers; for instance, the co-expression of IGF2BP3 and the lncRNA DDRMR is a diagnostic and prognostic marker in clear cell renal cell carcinoma (ccRCC) (Gu et al., 2021a). IGF2BP3 has been demonstrated to promote cancer progression by being stabilized by ncRNAs and stabilizing mRNA of downstream genes. The upregulation of IGF2BP3 in GC could be a prognostic biomarker associated with an advanced stage (Zhou et al., 2017). The upregulation of IGF2BP3 is associated with poor OS and an advanced stage of colon cancer, suggesting that IGF2BP3 can serve as a prognostic biomarker (Yang et al., 2020b).

The targets of m^6^A regulators could also have prognostic value. For instance, levels of m^6^A vary among the mRNAs of cancer-related genes, such as *MORC2* and *PARM1*, and might predict CRC prognosis (Zhang et al., 2021d). The above findings are all based on comparisons of tumor tissues and normal tissues; however, a recent study has revealed that m^6^A in the peripheral blood is an effective marker for assessing prognosis in patients with GC after surgery (Ge et al., 2020), providing a novel direction for the diagnosis and treatment of GI cancer.

### 3.2 m^6^A in gastrointestinal cancer therapy

m^6^A methylation contributes to clinical treatment approaches. Since m^6^A regulators can be affected by several small molecules and drugs, they may provide a basis for the development of potent therapeutic targets and tumor hallmarks (Nombela et al., 2021).

#### 3.2.1 Immunotherapy

Increasing evidence has demonstrated that m^6^A modification has critical functions in tumor-related immune processes, providing novel therapeutic targets based on the modulation of immune responses. m^6^A participates in the inhibition of innate immunity targeting circRNAs, which are suppressed by YTHDF2 in the cytoplasm (Chen et al., 2019b). m^6^A mediates not only innate immune responses but also adaptive responses (Paramasivam and Vijayashree Priyadharsini, 2020). For instance, YTHDF1 can lead to immune escape by binding to the mRNAs of lysosomal cathepsins, facilitating translation and suppressing cross-presentation of dendritic cells (DCs) (Han et al., 2019b). Increased transcription of CD40, CD80, and the TL4 signaling adapter Tirap is modified by METTL3 in DCs, leading to T cell stimulation (Wang et al., 2019c). T cell homeostasis has been shown to be maintained by the regulation of m^6^A targets such as IL-7, STAT5, and SOCS (Li et al., 2017b). Song et al. explained the restriction of natural killer cells in anti-tumor immunity, which enhances cancer development via the reduction of SHP-2 m^6^A modification by decreased METTL3 (Song et al., 2021), indicating that m^6^A has an important role in homeostasis and the infiltration of NK cells. In addition, m^6^A methylation can regulate the immune response in GC by interferon modulation (Zhang et al., 2019b).

In addition to its involvement in the regulation of immune cells, m^6^A also activates other key cells that are crucial in cancer immunity. It has been observed that m^6^A modification is correlated with the tumor microenvironment (TME), due to its modulation of hypoxia, metabolic dysregulation, immune escape, and chronic inflammation (Gu et al., 2021b; Li et al., 2022a), where T-cell transport varies significantly, forming different patterns of infiltration in GC and providing a novel system for evaluating prognosis and guiding immunotherapy (Zhang et al., 2020b). Immune cell infiltration and T-cell associated immune responses are restrained when WTAP is upregulated in GC (Li et al., 2020). Similarly, Bai et al. have reported that the upregulation of YTHDF1 in GC limits the induction of dendritic cell recruitment and infiltration of CD4^+^ and CD8^+^ T cells, repressing anti-tumor immunity (Bai et al., 2022). METTL14 is downregulated in macrophages of CRC, promoting tumor progression; this reveals a potential relationship between these cells and the infiltration of surrounding CD8^+^ T cells in the TME. Accordingly, METTL14 might be a new target of CRC immunotherapy (Dong et al., 2021). We can manipulate m^6^A modification of immune cells for improving immunotherapy outcomes in GI cancer patients. A research team has utilized nanoparticle-encapsulated YTHDF1-siRNA to enhance anti-tumor immunity in CD34 humanised CRC mouse model (Bao et al., 2023). Another study demonstrates that depletion of METTL3 or METTL14 could increase the sensitivity of anti-PD-1 therapy by supporting the function of cytotoxic tumor‐infiltrating CD8 T cells (Wang et al., 2020e). These studies collectively illustrate the potential link between m^6^A regulators and the efficacy of immunotherapy in GI cancer, and suggest new therapeutic targets.

The levels of m^6^A regulators could be used to predict immune features of the TME (Gu et al., 2021b). Based on a principal component analysis, an m^6^A score associated with the TME phenotype was established and showed predictive value for the anti-PD-1/L1-based immunotherapy response in GC (Zhang et al., 2020b). In colon cancer, an m^6^Sig scoring system for quantifying the levels of m^6^A, influenced by m^6^A phenotype-related genes, is correlated with immune infiltration and immune responses (Chong et al., 2021). In addition, a reduction of METTL3 in macrophages inhibits the efficacy of Programmed Cell Death (PD-1) blockade therapy (Yin et al., 2021). However, Wang et al. recently found that in pMMR-MSI-L CRC tumors with low mutational burdens, the depletion of METTL3 or loss of METTL14 stimulates the secretion of CXCL9 and CXCL10 and induces METTL3/14-related *STAT1* and *IRF1* mRNA stability by YTHDF2, improving the response to anti-PD-1 therapy (Wang et al., 2020e). These two contradictory effects of METTL3 on anti-PD-1 treatment might be attributed to differences among cancer types, as well as distinct regulatory mechanisms.

#### 3.2.2 Therapy resistance

Resistance to radiotherapy, chemotherapy, immunotherapy, and molecular targeted therapy is an urgent problem. Given that the microenvironment around the solid tumor is complex, radiotherapy resistance is associated with multiple characteristics, such as DNA damage, reduced apoptosis, arrested cell cycle, and dysfunctional mitochondria in GI cancer cells. Based on (5-fluorouracil) 5-FU, chemotherapy regimens for GI cancer display the limitation due to drug resistance, which causes relapse after standard chemotherapeutic courses. Although patients’ specificity is considered by immunotherapy strategy like anti–PD-1/PD-L1 immune-checkpoint inhibitor (ICI) treatment, mutation patterns such as mismatch-repair- proficient (pMMR) and microsatellite-stable (MSS) of CRC lead to not respond to immunotherapy. Several mechanisms underlying therapeutic resistance have been reported, such as multi-drug resistant genes, epigenetic changes, DNA damage repair, and cancer stem cells (Holohan et al., 2013). In addition to applications for the exploration of novel immunotherapy drugs, m^6^A plays an important role in therapy resistance (Zhuang et al., 2023). m^6^A mediates therapy resistance by regulating the drug transportation, autophagy, DNA damage repair, and TME remodeling (Lin et al., 2022). As mentioned above, METTL3 is highly expressed in GC via the METTL3/miR-17-92 pathway and is related to an elevated sensitivity to everolimus (Sun et al., 2020). However, Li et al. reported that METTL3 is elevated in CD133+ GC stem cells and the recruitment of YTHDF1 to the 3′UTR of *PRAP1* mRNA stabilizes PRAP1, mediating oxaliplatin resistance (Li et al., 2022b). Additionally, m^6^A methylation of human Polycomb 3 (CBX8) increases chemoresistance in colon cancer by maintaining the stability of *CBX8* mRNA (Zhang et al., 2019c).There is evidence suggesting that chemotherapeutic resistance in CRC relies on METTL3-mediated Sec62 expression (Liu et al., 2021). METTL3 is also upregulated in tumor-associated macrophages (TAMs) in patients with CRC with oxaliplatin resistance and downregulates TRAF5, inhibiting necroptosis (Lan et al., 2021), similar to its role in GC. In subsequent work, Pan et al. explored the mechanism by which m^6^A contributes to 5-fluorouracil (5-FU) resistance and found that exosomal miR-181d-5p derived from cancer-associated fibroblasts (CAFs) is promoted by METTL3 in CRC cells, inhibiting neurocalcin δ (NCALD) and mediating resistance to 5-FU (Pan et al., 2022). These data indicate that METTL3 mediates different drug resistance mechanisms for different chemotherapy regimens in GC, providing a new target for individualized treatment and overcoming drug resistance. Furthermore, Chen et al. found that increased YTHDF1 can improve cisplatin resistance in colon cancer by increasing glutaminase (GLS) translation (Chen et al., 2021c).

In addition to chemotherapy resistance, m^6^A is involved in resistance to molecular targeted therapy. In resistant leukemia cells treated with tyrosine kinase inhibitors, the overexpression of FTO elevates mRNA stability and promotes tumor cell survival, providing a target for inhibition of drug resistance (Yan et al., 2018). Resistance to cetuximab, which targets epidermal growth factor receptor (EGFR), is related to the upregulation of pleckstrin homology-like domain, family B, member 2 (PHLDB2), whose mRNA might be methylated by METTL14 (Luo et al., 2022). These results suggest that the abnormal expression of m^6^A regulators mediates drug resistance or that various treatments alter their expression patterns and thus result in resistance. Given that most m^6^A regulators tend to enhance the resistance of GI cancer, selective small-molecule inhibitors of these regulators, which combines other therapies such as chemotherapy, might be applied in the clinic.

## 4 Conclusions and future prospective

As a major focus of recent research, m^6^A is involved in various aspects of cancer biology, including development and cancer-related metabolism. The feasibility of high-throughput sequencing and other detection techniques has improved our understanding of the critical functions of m^6^A modification in controlling cancer cell phenotypes and gene expression, particularly by the post-transcriptional regulation of mRNAs and ncRNAs. In this review, we comprehensively summarized recent progress on the mechanisms of m^6^A regulation in GI cancer from the perspectives of related coding and non-coding RNAs and the potential impact of m^6^A on the efficiency of GI cancer therapy. From the existing researches, we have found that m^6^A regulates tumor growth and progression, and we can predict that m^6^A regulators can be associated with prognosis of GI cancer patients, which provides a new idea for future GI cancer diagnosis and therapy. Previous studies have revealed that ncRNAs are involved in cancer development, including tumor proliferation, invasion, metastasis, and carcinogenesis, and are mutually regulated (Yi et al., 2020). However, further research related to the specific regulatory mechanism underlying the effects of m^6^A, such as its direct function on RNAs, is still in a preliminary stage in GI and other cancers. Relationships between m^6^A factors and their targeted RNAs provide a novel direction for clinical diagnosis and treatment. However, the vast majority of published studies explore the m6A regulatory factors as tumor prognostic markers and reflect the value of the treatment effect. Meanwhile, almost no studies address ncRNA as a potential diagnostic biomarker. This might be due to the abnormal expression of these molecules, which can be detected in a wide variety of cancers; in addition, the regulatory mechanism in each cancer is still in the exploratory stage. Additionally, it is unclear whether the risk factors of GI cancer, such as infection of *Hp*, diabetes, and aging leading to carcinogenesis are due to the change of the m^6^A level. As a result, its specificity as an early diagnostic marker is insufficient. Furthermore, future studies of m^6^A regulation should focus on systematically combining concrete protein, RNA modification, and signaling pathway data. The effect of small molecular specific inhibitors of m^6^A are still observed in mouse models, and has not been reported clinically. Exploring effective inhibitors targeting m^6^A regulators and combining them with the existing drugs will provide a new window for GI cancer treatment.
